# Peer support for CKD patients and carers: overcoming barriers and facilitating access

**DOI:** 10.1111/hex.12348

**Published:** 2015-02-03

**Authors:** Francesca Taylor, Robin Gutteridge, Carol Willis

**Affiliations:** ^1^ NIHR CLAHRCSchool of Health and Population SciencesUniversity of BirminghamBirminghamUK; ^2^ Faculty of Education Health and WellbeingUniversity of WolverhamptonWolverhamptonUK; ^3^ Department of Renal MedicineHeart of England Foundation TrustBirminghamUK

**Keywords:** barriers, chronic kidney disease, facilitation, participation, peer support

## Abstract

**Background:**

Peer support is valued by its users. Nevertheless, there is initial low take‐up of formal peer support programmes among patients with chronic kidney disease (CKD), with fewer patients participating than expressing an interest. There is little evidence on reasons for low participation levels. Few studies have examined the perspectives of carers.

**Objective:**

To explore with CKD patients and carers their needs, wants and expectations from formal peer support and examine how barriers to participation may be overcome.

**Methods:**

Qualitative interviews with a sample of 26 CKD stage five patients and carers. Principles of Grounded Theory were applied to data coding and analysis.

**Setting:**

Six NHS Hospital Trusts.

**Results:**

Whilst informal peer support might occur naturally and is welcomed, a range of emotional and practical barriers inhibit take‐up of more formalized support. Receptivity varies across time and the disease trajectory and is associated with emotional readiness; patients and carers needing to overcome complex psychological hurdles such as acknowledging support needs. Practical barriers include limited understanding of peer support. An attractive peer relationship is felt to involve reciprocity based on sharing experiences and both giving and receiving support. Establishing rapport is linked with development of reciprocity.

**Conclusions:**

There is potential to facilitate active uptake of formal peer support by addressing the identified barriers. Our study suggests several facilitation methods, brought together in a conceptual model, including clinician promotion of peer support as an intervention suitable for anyone with CKD and their carers, and opportunity for choice of peer supporter.

## Introduction

A diagnosis of chronic kidney disease (CKD) can be devastating for people, creating physical and emotional life changes with accompanying difficult social and psychological challenges.^1^, ^2^, ^3^ Peer support is based on the premise that those who have been in a similar position are best placed to help support their peers with both the experience and treatment of this disease. It is recognized as an important component of quality pre‐dialysis care and in preparing patients and carers for renal replacement therapy (RRT).^4^ Additionally, peer support can facilitate utilization of home haemodialysis (HHD).^5^

Peer support is a multifaceted concept whose meanings and characteristics depend on the context of use. It may be delivered in a range of different combinations of mode, duration, personnel and intended outcomes. Within the context of care for long‐term conditions, peer support programmes generally aim to provide sharing of emotional and practical experiences, and information, among patients with the same chronic condition.^6^, ^7^, ^8^ Peer support can be informal or formal, where the support is offered by a purposefully trained patient or carer. Formal peer support is actively promoted in current health policy.^9^

Formal peer support interventions have been used for a range of different long‐term conditions, as well as CKD. In terms of improved physical and mental health outcomes, the evidence is inconclusive. From studies of formal peer support among patients with CKD, reported outcomes are generally positive, but the available evidence is insufficiently robust.^8^, ^10^, ^11^, ^12^, ^13^ More substantive evidence from studies among patients with long‐term conditions in general shows weak beneficial outcomes,^14^, ^15^, ^16^, ^17^, ^18^, ^19^, ^20^ mixed results^21^, ^22^ or no positive impacts.^23^

Yet formal peer support is well received by its users among patients with CKD. Of particular value are help in adjusting to their illness; being able to talk to someone who can listen and empathize; gaining confidence and more sense of control; and having access to practical information based on the lived experience of treatment.^8^, ^10^, ^12^ Similar findings have been reported in peer support programmes for other long‐term conditions, notably cancer and mental health.^17^, ^19^, ^20^, ^24^

Peer support can also be beneficial in helping patients with CKD make treatment choices and alleviate fears about possible therapies.^2^, ^25^, ^26^, ^27^ Hearing about a particularly good or bad experience with a particular dialysis modality can even cause patients to change an initial decision.^28^ Patients may approach the peer encounter with the intention of actively seeking specific information to help them reach decisions about treatment.^20^

Despite peer support being valued by its users, there appears to be initial low take‐up of formal peer support among patients with CKD, with fewer patients participating than expressing an interest.^10^, ^29^ Formal peer support programmes for other long‐term conditions have recognized similar issues.^14^, ^30^, ^31^, ^32^ Patients from identified socio‐economic groups (older, male, lower educational attainment, lower social groups) are under‐represented.^14^, ^30^, ^31^, ^32^ In order to widen participation, it may be important to understand whether this low take‐up is the result of misconceptions, lack of access or encouragement, and/or surmountable barriers.^31^ Although carers often have a significant role in the health care of patients with CKD, particularly HHD, few studies have examined the needs or perspectives of carers.

Many UK Renal Units have recently set up or are planning formal peer support services, requiring investment of clinician time and resources. Therefore, a Renal Network already in the process of establishing a formal peer support programme for patients with CKD and carers (Table 1), and a colocated Health Innovation Education Cluster, decided to conduct a research study to better understand the issues impeding participation. The aims of the study we report here were to: explore with CKD patients and carers their needs, wants and expectations from formal peer support; examine how barriers to participation may be overcome; and recommend service improvements.

**Table 1:** Local Renal Network's peer support model

Opportunity to have a one‐to‐one, confidential chat over the telephone with an experienced patient Short‐term emotional, practical and/or social support based on one or two conversations, not a longer‐term relationship Available to all patients with CKD but with a focus on use, ○ when first diagnosed ○ when making decisions about treatment therapy ○ when considering whether to go on the transplant list ○ when considering whether to undergo live kidney transplant Complementary to care and education received from the patient's renal health‐care team Provided by volunteer patients and carers who have undergone Criminal Records Bureau checks and training for the role of peer supporter Peer supporters recruited through use of posters, local renal patient and carer forums, local Kidney Patient Association, the Renal Network's website, and letters from clinicians to patients identified as suitable Database created with details of all trained peer supporters across the Network – including age, gender, treatment type, working status, ethnicity and language spoken – to enable matching of a suitable peer supporter with each patient Service set‐up and managed by a Network clinical champion and dedicated staff in each Hospital Trust Accessed by patient self‐referral or referral by a clinician

## Methods

Patients with CKD and carers were recruited to the study from six NHS Hospital Trusts. Ethical approval was received from the Local Committee of the National Research Ethics Service Committee (12/EM/019) and the host University (ERN‐12‐0334).

### Design

A qualitative study design was employed to enable insights into patients' with CKD and carers' attitudes and opinions, and to better understand the social actions and processes involved in formal peer support.^33^ Patients and carers were interviewed individually in their home or Renal Unit, dependent on participant choice. The interviews lasted between 45 and 90 min. Semi‐structured questions with supplementary prompts (Table 2) were used to allow the key areas of research interest to be explored without being overly prescriptive about content and direction.^33^ In each interview, participants were read a description of peer support: ‘Peer support is where patients and carers with experience of chronic kidney disease help other kidney patients and carers facing similar situations. It is additional support to that provided by your doctors and nurses'. All interviews were digitally recorded, professionally transcribed in full and the transcripts checked against recordings.

**Table 2:** Semi‐structured interview question and interview prompts (example)

Question: How could peer support be designed to best suit you?
Question prompts: How would you like to find out about peer support? What would you want to know? How would you prefer to access peer support – face‐to‐face, by telephone, on the Internet, in a group, one‐to‐one? What qualities would you like your peer supporter to have? When would it be most useful for you to use peer support? How long for?
Exploratory prompts: Why do you feel that way? Can you tell me a little more about that? Why is that? Anything else you can think of?

### Recruitment

Inclusion criteria specified consenting adult patients – and adult carers of patients – with CKD stage 5, established kidney failure. Sampling was purposive, designed to provide maximum diversity of socio‐economic group, position along the CKD stage 5 pathway, and dialysis treatment type.

Within each Renal Unit, a member of staff acted as gatekeeper. They identified patients with CKD and carers who met study eligibility criteria and made the initial enquiry about participation. Patients and carers expressing interest were given a study Information Sheet and asked whether they were willing to have their contact details passed to a researcher. A total of 48 patients and carers were contacted by a researcher and 27 (56%) agreed to be interviewed. One patient withdrew before interview due to ill health. All access and consent processes complied with ethical principles.^34^ Consent was considered an on‐going process. At the end of each interview, interviewees were offered the option of commenting on and amending their interview transcript.

### Participants

There were 26 participants, 15 patients and 11 carers. Details of the study participants are shown in Table 3. Patients ranged in age from 36 to 77 years and carers from 52 to 67 years. There were 10 male and 16 female respondents. All participants were patients, or carers of patients, with established kidney failure and at different positions along the CKD stage 5 pathway. Most participants were not working, but four carers and two patients worked full‐ or part‐time. All but three respondents were married. A total of 10 participants said they had no educational qualifications, and 16 participants reported having GCSE/‘O' level qualifications or above. Regrettably, all interviewees were White British apart from one British Asian.

**Table 3:** Details of study participants (self‐reported)

Designation	Gender	Age (years)	Location	Marital status	Working qualifications	Educational qualifications (GSCE/O level +)	Current therapy
Carer	F	59	Rural	Married	Working p/t	Yes	Training HHD
Carer	F	57	Rural	Married	Not working	No	HHD (8 months)
Patient	F	46	Urban	Married	Not working	Yes	Awaiting transplant
Patient	F	36	Urban	Single	Not working	Yes	HHD (3 months)
Carer	F	60	Urban	Married	Working f/t	Yes	HHD (3 months)
Patient	F	62	Rural	Married	Working p/t	Yes	Pre‐dialysis
Carer	F	56	Urban	Married	Not working	No	HHD (7 months)
Carer	F	52	Urban	Married	Not working	Yes	HHD (3 months)
Patient	F	46	Urban	Married	Not working	Yes	Awaiting transplant
Patient	F	50	Rural	Married	Not working	No	HHD (3 months)
Carer	M	57	Rural	Married	Working f/t	Yes	HHD (3 months)
Patient	M	72	Rural	Married	Not working	Yes	HHD (18 months)
Patient	M	70	Rural	Married	Not working	Yes	HD (12 months)
Patient	F	65	Urban	Widowed	Not working	No	HD (24 months)
Patient	F	76	Rural	Widowed	Not working	No	HD (30 months)
Carer	F	66	Rural	Married	Not working	No	PD (14 months)
Carer	F	67	Rural	Married	Not working	No	HHD (9 months)
Patient	M	65	Rural	Married	Working p/t	No	HHD (9 months)
Patient	M	38	Urban	Married	Working f/t	Yes	Transplant
Patient	M	77	Rural	Married	Not working	Yes	PD (18 months)
Carer	M	67	Urban	Married	Not working	Yes	Pre‐dialysis
Patient	F	63	Urban	Married	Not working	Yes	Pre‐dialysis
Carer	M	62	Urban	Married	Working f/t	Yes	HHD (3 months)
Patient	M	71	Rural	Married	Not working	No	PD (14 months)
Carer	F	64	Rural	Married	Not working	Yes	HD (12 months)
Patient	M	59	Rural	Married	Not working	No	Training HHD

None of the respondents had engaged in formal peer support, although all participants had some experience of informal peer support. Only two patients reported having been offered formal peer support, but both had declined the offer.

### Analysis

All interview data were coded and analysed using principles of Grounded Theory.^35^, ^36^ Transcripts from the first six interviews were read and re‐read by one researcher. Data were broken down using line‐by‐line coding and the codes clustered to identify initial categories based on ideas, issues and themes. The emerging codes and categories were discussed after scrutiny by a second researcher who had also read the transcripts. Together the researchers asked questions of the data to assist identification of category properties. For example, why did this participant experience occur? Who experienced these feelings?

Constant comparison was utilized with each data collection from further interviews compared with every other for similarities, differences and connections. Categories were refined and enhanced, some combined and others condensed or removed. This process was undertaken independently by one researcher supplemented by continuous collaborative discussion with the second researcher to reach consensus and confirm categories.^37^, ^38^

## Results

Following analysis four main categories were identified: perceived benefits of peer support over other sources of support; the peer support occasion; permission to engage; and the core category, an attractive peer relationship.

### Perceived benefits of peer support over other sources of support

Both patients and carers felt that peer support has specific attributes and benefits over and above existing support provided by family and friends. These perceptions were based on two contextual influences. First, informal peer support experience, involving patients and carers conversing with others in the same situation as themselves, generally as a result of incidental encounters at Renal Units. Second, the realization that existing relationships and support networks did not meet all their needs. It appeared that established kidney failure engenders an altered conception of self in relation to others; patients and carers mentioned having to adjust their lives and lifestyle and how this changed relationships with family members, friends and work colleagues. Some respondents reported a strong desire to protect and not ‘burden’ those people close to them with the reality of their feelings.

The anticipated informational benefits of peer support were attractive to many participants. Some emphasized the value of learning about the future course of their condition and treatment, and the impact on their lifestyle, to reduce uncertainty about their disease progression and feel more in control.

To learn, share experiences, you know, get an idea of what's coming up next. What should I expect, you know, if I encounter a problem? Should I be worried? It's just having that someone who's been through that before to be able to talk to. (Patient 7)

Several patients new to home dialysis mentioned the advantages of learning practical adaptive coping skills, for example aspects of needling. Other participants talked enthusiastically about gaining knowledge about how to address particular personal issues in relation to their illness and treatment.

General everyday things, sex and things like that… find out if they've been in that situation. (Carer 6)

Feelings of acceptance and understanding were also important benefits associated with peer support, the affirmation of shared emotional experiences providing the reassurance and comfort of not being alone. Whilst significant for both patients and carers, these benefits were especially salient for carers of patients newly on HHD. They frequently referred to feeling isolated in adjusting to their new role and responsibilities. Not only did they consider they put their partner's needs above their own, but so did their clinicians, family and friends.

On your own and isolated and you know you sort of think, I'm the one that's supposed to be doing everything, I'm supporting you, where's my support? (Carer 5)

Validation of personal feelings and behaviour was another strong motivator for interest in peer support. Most respondents wanted affirmation of the normality of their own experiences.

Talk to other people and see whether they're moving roughly down the same route that you are, or whether you are just, well whether you're better or worse, you know. It's just a matter of trying to think well is everything normal you know. (Patient 6)

Participants were also keen to make active comparisons with others in a similar position, to compare positively upwards not downwards. This stemmed from a desire to be guided to new possibilities and opportunities in managing CKD and its treatment. Respondents wanted vicarious encouragement that improvements were possible, and a role model, not someone imbued with pity.

In comparison with clinicians, peer supporters were thought to provide a ‘*truer*’, more rounded and insightful picture of what a particular RRT involves and how it feels. Whilst highly appreciative of the information and support provided by nurses and consultants, it was recognized they cannot provide the *‘real’* knowledge that comes from a patient's or carer's lived experience of CKD and its treatment.

Not because the medics are bad or anything, it's just because they've just not walked that journey in the same way. They've sort of walked alongside you and are more observing, whereas this is more living it. (Patient 7)

Talking to a patient or carer peer was considered a very different type of discussion to that between patient/carer and clinician. The latter was characterized as being more hierarchical and clinician‐led; conversations tending to be predominantly medical focused. By contrast, peer support discussions were viewed as less constrained and more between equals. There was less of a clinical perspective with more emphasis given to emotional, practical and lifestyle issues. The language used between peers was also viewed as different, discussions being more in layperson's terms.

### The peer support occasion

The value and relevance of formal peer support was not viewed as time specific. There appeared to be different ‘occasions’ across time and the CKD pathway when peer support might be appropriate. Whilst practical issues such as travel requirements, time available and health status have substantial influence, these peer support ‘occasions’ seemed primarily to be associated with a complex mix of emotional readiness and intensity of need.

Respondents explained how their emotional ‘mood’ or ‘frame of mind’ at particular points might inhibit or motivate response to a desire to talk with another patient or carer. Some participants described occasions when they had particularly pressing support needs, but recognized it would have been too difficult for them to discuss these issues with a peer at that moment. On other occasions, they felt psychologically more willing and able to engage with peers.

I think there's different stages you need it…Sometimes you just feel like I don't want to talk about it, I don't want to know…Sometimes it's you're overloaded with what's happening to you. (Patient 15)

The individuality of the peer support ‘occasion’ was clearly evident. For example, some participants thought peer support would have been beneficial when they first received a diagnosis of kidney disease, to help reduce the inevitable uncertainties about their condition, its future course and effect on their life. Others felt too overwhelmed by the shock and fear of the diagnosis to have the emotional capacity to talk with other patients or carers at that point.

Not at the initial diagnosis because you need to get to grips with that…but sort of within a few weeks. (Carer 4)

Similarly, several HHD patients and carers thought listening and talking to people already using HHD might have given them more confidence and assurance choosing the therapy. Others felt they did not know enough at the decision‐making stage to be able to ask relevant questions and would have preferred to use peer support after being on HHD for a few weeks.

### Permission to engage

The term ‘peer support’ did not always have meaning for participants. This unfamiliarity led several patients and carers to dismiss ‘peer support’ as not for them and deterred others. The term could also promote a sense of exclusion or stigma. Some respondents misinterpreted the term.

I wouldn't know really…It'd go over me. (Carer 3)

It's fear of the unknown. (Patient 14)

Until ‘peer support’ was explained, many respondents were unaware this was a descriptor that could be applied to the encounters and conversations they informally engaged in with patients or carers, in a similar situation to themselves.

That's what I do, but I didn't know I was doing it, the name for it. (Patient 12)

Furthermore, ‘peer support’ was judged a somewhat cold and unfriendly term by those for whom it was unfamiliar. Respondents thought it sounded professional and inflexible, not especially welcoming or accessible.

Acknowledging a need for support was difficult for many patients and carers in this study. Seeking support from people outside their personal networks was not concordant with their habits, self‐image and perceptions of how to handle illness. This dissonance posed a challenge to self‐esteem. Respondents expressed concern about being perceived as overly ‘needy’ or lacking social resources. One carer had the impression peer support was only for people with serious problems, ‘like the Samaritans or Alcoholics Anonymous’. A number of participants, in particular patients dialysing at home for several months, and their carers, intimated they would be more comfortable being the provider rather than the recipient of support; the role of helper was both more familiar and more attractive.

Projected to me is you are a needy person and I don't like that picture of myself. (Carer 7)

Fear of negative professional judgement was another barrier to engagement. Some pre‐dialysis participants worried that if they took‐up formal peer support, it might give clinicians the impression they did not have the ability to manage their chosen therapy. This was a particular worry for some patients and carers considering HHD. They did not want to undermine their desired projected image of being sufficiently independent and capable of managing the treatment themselves.

Even if they weren't judging you, I think you'd feel they were, well I would. They've trained me, they think I'm ready. (Carer 2)

A few interviewees were uneasy about self‐referral or self‐reporting an interest in peer support to clinicians, in case this might be interpreted unintentionally and negatively as a criticism of clinician‐based support.

Perceiving formal peer support intrinsically as a social event, some respondents worried about not knowing the norms, obligations or boundaries.. They also felt their self‐efficacy and social skills would not be sufficient. Concerns were expressed about being too shy, unconfident, not very sociable, unable to convey needs and preferring to listen rather than talk.

I'm not very sociable. I find it hard to talk to people I don't know so I'd find it difficult to be honest. (Patient 3)

In this context, a number of participants wanted their clinicians to confirm they were suitable for peer support, or to affirm it was acceptable for them to engage in peer support.

I think the nurses would be a great help because they obviously know what sort of people the patients are and they can perhaps encourage them. (Patient 14)

### An attractive peer relationship

Whilst many participants perceived formal peer support as a valuable opportunity to learn from more experienced patients with CKD and carers, there was general resistance to the notion of being a passive recipient. A more reciprocal and mutual exchange was sought that would involve a balance of support and giving. Several interviewees felt an unequal exchange might generate an uneasy ‘support debt’ that would eliminate the potential for ease and comfort in the transaction.

I wouldn't feel right if they were just, somebody was just giving me hundred percent and I wasn't giving them something back…If I thought it was one‐sided I wouldn't even do it. (Carer 2)

Respondents recognized that formal peer support exchanges would inevitably have some imbalances and be different to most social interactions; for example, they acknowledged that peer supporters would need to be trained as good listeners. Nonetheless, many participants considered reciprocity and mutuality as key to encouraging their participation and important in preserving their dignity and self‐esteem.

I can accept it on an equal basis. I can offer somebody support and I can accept their support emotionally, I can deal with that. But for me to need support, emotional support, and not give anything back would be very hard. (Patient 5)

### Building rapport

Establishing good rapport with formal peer supporters was identified by the majority of patients and carers as a significant factor for enabling positive encounters. If rapport is lacking, it was widely assumed the relationship would not work. For most participants, rapport was closely aligned with creating the safe, trusting and empathetic ‘*place*’ where sharing and exchange could take place; the right emotional context for honesty and disclosure, especially of personal issues.

I think you always want to make that contact with someone before you trust. (Carer 6)

If you build up like a rapport with people I could probably tell them things, how I'm feeling, that I don't want to burden (carer) with. (Patient 3)

When asked to describe peer supporter characteristics they felt would help build rapport, most respondents judged commonality of disease and therapy experience to be insufficient. A small number of interviewees felt similar socio‐economic circumstances were important. In general though, the qualities needed were considered more personal attributes such as manner, presentation, sensitivity and communication style, rather than clearly definable skills or competencies. Using their informal peer support experience as a benchmark, some respondents described using a natural filtering process among patients or carers they encountered that led them to engage selectively. Almost all interviewees gave an account of patients or carers with whom they formed no meaningful bond or actively disliked. Interestingly, several respondents thought the ‘right person’ with whom they could establish rapport might vary over time, depending on their emotional and physical states, and particular needs.

At least some face‐to‐face contact was widely perceived to be necessary for rapport to be established. For example, two keen users of internet kidney patient forums said they often wanted supplementary face‐to‐face contact with peer supporters. This enabled them to establish a stronger one‐to‐one connection, allowing more significant discussion. Similarly, whilst recognizing the practical advantages of telephone‐based peer support, some respondents were nonetheless unenthusiastic. They anticipated telephone contact being somewhat cold and impersonal and felt it would be difficult to build rapport with an unseen person.

I don't think I could just, you know, have a one to one (on the phone)…if it's personal things. (Patient 2)

### Choice and control

Having choice and control in relation to certain aspects of the timing and delivery of formal peer support were important considerations for some interviewees. A need for autonomy in relation to their peer support was expressed most strongly among patients and carers who were either training to use, or already on, home dialysis therapies. This appeared consistent with their motivations for choosing home therapies: greater choice, control and ownership over their dialysis regime.

Being able to take a key role in choosing their own peer supporter/s was viewed as especially important, mainly because of the desire to ensure rapport is established as a precursor to shared exchange and reciprocity. Having the opportunity to choose when formal peer support takes place was another critical issue for some interviewees.

Preferences for the format and delivery of formal peer support varied considerably, and there was a strong desire for choice. Some respondents described wanting only to participate in peer support if it involved group contact. They felt this would make it easier and more comfortable for them to choose their level of engagement. Others expressed a preference for one‐to‐one meetings, for a variety of different reasons. These included the following: feeling too shy to engage in group support with unfamiliar people; perceiving it easier to build rapport with a single peer supporter; being more comfortable to discuss personal, intimate issues; and a belief they could have more control over the subject matter discussed. Several participants said they could only imagine themselves engaging in one‐to‐one peer support that was face‐to‐face because they could not envisage being able to build rapport and mutual understanding by any other means. There were other patients and carers who perceived advantages in one‐to‐one telephone encounters, mainly because of the convenience: no need to travel and fitting more easily into their daily life. Some interviewees felt choice of format would better suit their needs as these were likely to change over time.

Different people need different things at different times. (Patient 15)

## Discussion

Although results cannot be generalized from this small‐scale qualitative study, it has achieved a better understanding of CKD stage 5 patients' and carers' needs, wants and expectations from formal peer support. The study has identified some actual or perceived barriers to take‐up of formal peer support and proposed approaches for overcoming these. It both affirms existing literature and offers additional illumination.

Based on their use of informal peer support and experience of unmet needs, almost all study participants – carers as well as patients – clearly recognized a range of attributes and benefits associated with peer support. The main perceived benefits accord with the positive effects of participation identified in existing literature: feeling more in control;^10^, ^39^ reduced uncertainty;^40^ sense of empowerment;^19^, ^41^ being understood and accepted;^10^, ^42^ belonging and community centred;^43^ less isolated;^43^, ^44^ sense of normality;^17^ and the potential for more positivity and new possibilities.^14^, ^19^

**Figure 1:**
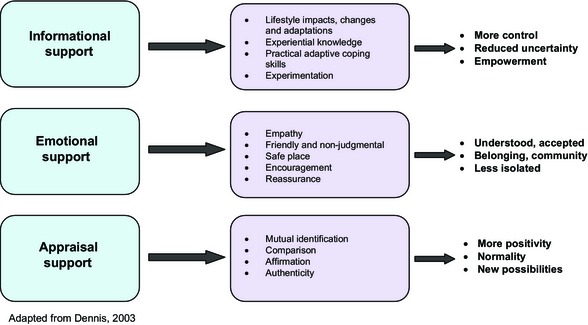
Perceived attributes and benefits of peer support.

The core mechanisms identified by Dennis^7^ as underpinning how peer support operates – ‘informational’, ‘emotional’ and ‘appraisal’ – were confirmed by this study and provide a helpful framework by which to examine and explain the identified attributes and benefits (Fig. 1).

**Figure 2:**
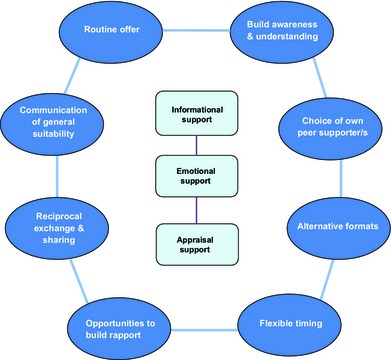
Conceptual model for facilitating access to formal peer support.

The merits of peer support for people with other long‐term conditions have long been recognized; the value of mutual support for patients with rheumatoid arthritis was identified by Bury over 30 years ago.^45^ Nonetheless, stimulating take‐up of formal peer support programmes remains problematic. This study has shown that whilst informal peer support might occur naturally and is welcomed, a range of emotional and practical barriers inhibit motivation to take‐up more formalized support. Addressing these issues should help facilitate the active uptake of formal peer support by patients with CKD and carers, with some transferability to peer support programmes for other long‐term conditions. Our study suggests several facilitation methods, brought together in a conceptual model that builds on Dennis' framework (Fig. 2).

Receptivity to formal peer support can vary across time and the disease trajectory and appears to be associated with emotional readiness; patients and carers needing to overcome complex psychological hurdles such as acknowledging the need for support. For a number of respondents, the notion of seeking support from people outside their personal networks was unfamiliar territory which challenged their self‐image and perceptions of how to manage illness. Health professionals can exert considerable positive or negative influence over patients' access to formal peer support^10^, ^30^ and might therefore consider offering greater ‘permission to engage’, promoting peer support as an intervention suitable for anyone with CKD and their carers, not just those who are ‘needy’.

Interestingly, the two patients in this study who had been offered and declined to take‐up formal peer support both associated their rejection with inappropriate timing. One patient explained that when peer support was proposed, they felt burdened with adjusting to their diagnosis and the implications of their illness, as well as handling the demands of being a wife and mother. Another patient described the offer of peer support as coming when he was just starting treatment and feeling insufficiently experienced to be able to discuss practicalities with a peer. These responses are congruent with findings from the literature indicating that timing of the peer support offer can be a factor influencing levels of participation.^46^, ^47^ Flexibility of peer support provision is therefore needed across the CKD stage 5 pathway.

Practical barriers to the take‐up of formal peer support included limited awareness and comprehension. The term ‘peer support’ was not universally understood, so in communications there may be a need for clearer definitions and explanatory information; emphasizing how peer support encounters enable experiences to be shared and exchanged.

Our findings revealed the importance of perceived reciprocity in the peer support relationship. Both patients and carers rejected the notion of a one‐way gift of help. This may be a reflection of the majority of study participants being ‘expert’ patients and carers, self‐caring on home dialysis therapies. Nevertheless, similar views were expressed by some pre‐dialysis patients and carers.

The value of reciprocity has been much emphasized in the literature on peer support, particularly in mental health services. Reciprocity is based on the opportunity for sharing experiences, both giving and receiving support, and for building a mutual and synergistic understanding that benefits both parties and is integral to a positive peer relationship.^6^, ^19^ Sustaining the attractive egalitarian aspects of peer support, reciprocity also helps avoid reproduction of traditional power hierarchies.^47^ It has been suggested that reciprocity is more likely to develop where there is minimal social distance, shared interest and commonalities in life‐experiences.^46^ Good ‘matching’ of peer and peer supporter has become a feature of several formal peer support programmes with clinicians providing a brokerage role, ‘matching’ on the basis of characteristics such as relevant treatment experience, gender, age group, ethnicity, family circumstances and employment status.^10^

Participants in this study linked establishing rapport with the development of reciprocity. To establish rapport more effectively, they wanted to be involved in choosing their own peer supporter. This was particularly important for patients (and their carers) choosing HHD as their modality and may perhaps indicate a desire for greater control over their illness and treatment, in line with Leventhal's model^48^ Hughes *et al*.^10^ also raised questions about the value of ‘matching’ patients with their peer supporter and the effects of this as well as other ‘brokerage’ aspects on the peer support relationship. Creating opportunities for rapport to be built with potential peer supporters should be considered, including initial face‐to‐face meetings. This respondent group also expressed doubt about the potential for rapport to be established by telephone peer support alone. However, evidence suggests that in practice, rapport can be rapidly built through this medium.^10^

In considering these findings, some limitations of the study sample should be borne in mind. Although the study endeavoured to employ a purposive sampling technique to provide maximum diversity, the sample obtained was predominantly white and female, with few representatives of people least likely to participate in formal peer support. In particular, there was a lack of ethnic diversity. Also only some of the recruited sample were pre‐dialysis, most being on RRT. Use of renal staff as gatekeepers may have resulted in recruitment of a convenience sample rather than the intended purposive sample.

Several recommendations arising from the study have been used by the local Renal Network to refine and develop their formal peer support programme and design how this is introduced and promoted to patients and carers. The availability and suitability of peer support for both patients with CKD and their carers is now highlighted in communications. Greater prominence is given to feedback on the benefits of peer support experienced by users. Also patients and carers now have the opportunity to be matched with a peer supporter of their choice.

The local Renal Network explored with patient and carer representatives use of alternative terms to ‘peer support’ that might be better understood. It has proved difficult to find a replacement. However, promotional material about the peer support programme has been amended to include a clearer definition of peer support and be more welcoming and friendly in tone.

There is now emphasis on formal peer support as a routine offer suitable for anyone with CKD and their carers. Clinicians also feel more able proactively to promote the peer relationship as an enabling experience of mutual sharing, exchange and support.

## Source of funding

This study was funded by the West Midlands Central Health Innovation and Education Cluster (WMC HIEC). Francesca Taylor is supported by the National Institute for Health Research (NIHR) Collaboration for Leadership in Applied Health Research and Care for West Midlands initiative (CLAHRC WM).

## Conflict of interest

None.
